# The role of patient positioning in surgical fixation of the calcaneus fractures using the lateral extensile approach

**DOI:** 10.1007/s00590-023-03829-y

**Published:** 2024-01-24

**Authors:** Hammam Kayali, Isam Moghamis, Mohammed Radi, Omar Baroudi, Ashraf Hantouly, Ahmad Toubasi, Mazen A. Foodoul, Mohammed Alkhayarin, Ghalib Ahmed

**Affiliations:** 1 Department of Orthopaedic Surgery, Surgical Specialty Center, Hamad Medical Corporation, Doha, Qatar; 2 School of Medicine, University of Jordan, Amman, Jordan

**Keywords:** Calcaneal fracture, Lateral extensile approach, Prone position, Lateral position, Calcaneus fixation

## Abstract

**Introduction:**

Calcaneus is the most commonly fractured tarsal bone. Open reduction and internal fixation of the displaced intra-articular fractures is considered the gold standard treatment. The lateral extensile approach is the most commonly used approach, and usually, the patients are kept in lateral decubitus position. Recent study has descried calcaneus fracture fixation utilizing the lateral extensile approach with the patient in prone position. The aim of this study was to compare the postoperative radiological outcome, reoperation rate, operative and anesthesia time, infection and the wound complications rate between the two groups.

**Methods:**

The data of 49 adult patients with unilateral closed calcaneus fracture underwent open reduction and internal fixation using lateral extensile approach were collected. Postoperative Bohler’s, Gissane angles and complications rate were compared between the two groups.

**Results:**

A total of 49 patients were included. Lateral position was utilized in 26 patients (53.1%), while 23 patients (46.9%) were operated in prone position. Majority of the patients were males 87.8% (43 patients), and the mean age of the patients was 31.12 ± 7.50. The most commonly mechanism of injury was fall from height in (91.8%) of the patients.

The mean preoperative Bohler’s angle was 9.33 ± 13.07 and increased to 22.69 ± 9.15 postoperatively. The mean preoperative angle of Gissane was 130.45 ± 26.98 whereas it was 124.76 ± 17.20 postoperatively. The mean postoperative Bohler’s angle and angle of Gissane were significantly higher among patient who underwent fixation in lateral position (25.88 ± 6.62, 137.15 ± 11.17) when compared to the prone one (19.09 ± 10.35, 110.74 ± 10.81). There was no significant difference between the two groups regarding the reoperation rate (*p* 0.947), infection (*p* 0.659, operative time (p 0.688), anesthesia time (*p* 0.522) and wound complications (*p* 0.773).

**Conclusion:**

Surgical restoration of the Bohler’s and Gissane’s angles with the patient placed in the lateral decubitus position remains superior to the prone position with no difference in the complication rate between the two groups.

## Introduction

Calcaneus is the most commonly fractured tarsal bone and accounts for approximately 60% of all tarsal bone fractures and 2% of all bone fractures [1, 2]. Anatomical characteristics of the calcaneus such as loose trabecular bone with thin cortices and its position in the hindfoot make it susceptible for fractures [2, 3]. These fractures are mainly occupational injuries that occur secondary to a fall from height in young laborer men [4–9]. Controversy on the treatment of calcaneus fractures remains, as several different operative and non-operative surgical strategies exist [10–12]. However, open reduction and internal fixation has been considered the gold standard treatment for intra-articular displaced calcaneus fractures, as it generally provides good functional outcomes and the ability to anatomically restore the subtalar joint [4].

Several surgical approaches have been described previously in the literature, with the extended lateral approach being the mostly used approach [11, 13–15]. Usually, patients undergoing surgical treatment with lateral extensile approach are placed in the lateral decubitus position [16]. A recent study by Hasan et al. has described the fixation of calcaneus fracture in prone position [17].

Up to our knowledge, there have been no studies in the literature comparing the effect of lateral versus prone position in the surgical management of calcaneus fractures.

The aim of this study was to investigate the effect of patient position on the postoperative radiological outcome and the complications rate in patients undergoing surgical fixation of the calcaneus utilizing the lateral extensile approach.

## Methodology

### Study design

This retrospective cohort study was conducted at an academic level I trauma center, and it was approved by the institutional review board. All the medical records, preoperative and postoperative radiographs of the surgically treated calcaneus fracture between [2006 and 2011 + 2015 and 2016] were reviewed to identify the eligible patients. The study compared the prone versus the lateral position for open reduction and internal fixation of displaced calcaneus fracture utilizing the L-shape extensile approach. The primary outcome was the postoperative Bohler’s angle. The secondary outcomes were as follows: Angle of Gissane, operative time, anesthesia time, infection rate, reoperation rate, and wound complications. Sanders’ classification was used to describe the fractures.

### Eligibility criteria

The inclusion criteria were patients above the age of 18, who sustained a unilateral closed calcaneus fracture Sanders II or III, that underwent open reduction and internal fixation using the L shaped extensile approach. The exclusion criteria were as follows: Patients below the age of 18, Sander I and IV, bilateral injuries, sinus tarsi approach, closed or open reduction and K-wire pinning, open injuries, incomplete documentation and lack of postoperative X-rays.

### Data analysis

Categorical variables were presented as counts and percentages while continuous variables were interpreted as mean, standard deviation and range. The differences in the characteristics and the outcomes of the patients operated in the lateral and prone positions **were** done using Chi-square test and T test for categorical and continuous variables, respectively. Variables that were significant in the Chi-square and T test were subsequently tested in the **multivariable logistic regression** to test the association between patients position and the outcomes of interest. Any test with a *P* value < 0.05 was considered significant. The data analysis was done using IBM-SPSS v.25.

## Results

### Participants

A total number of 110 patients were identified in our database search, 61 of who were excluded, as they did not meet the eligibility criteria. The total number of the included patients with surgically treated calcaneus fractures was 49 patients. Lateral position was utilized in 26 patients (53.1%), while the rest were operated in prone position.

Majority of the patients were males 87.8% (43 patients) with a mean age of 31.12 ± 7.50. Falling from height was the most commonly mechanism of injury (91.8%). Sanders Classification of the fracture among the included patients showed that 61.2% of the fractures were Type III and the rest were Type II. Infections occurred only in 2.0% of the patients. Furthermore, reoperation and wound complications occurred in 4.9 and 8.3%, respectively. The mean preoperative Bohler angle was 9.33 ± 13.07 while it was 22.69 ± 9.15 postoperatively. The mean preoperative angle of Gissane was 130.45 ± 26.98 whereas it was 124.76 ± 17.20 postoperatively. The characteristics of the included patients are described in Table 1.

**Table 1:** Demographic characteristics of participants

Variable	Response	Frequency (*n* = 49)	Percentage (%)
Sex	Male	43	87.8
	Female	6	12.2
Mechanism of injury	FFH	45	91.8
	Others	4	8.2
Laterality of the fracture	Left	23	46.9
	Right	25	51.0
	Bilateral	1	2.0
Sanders classification based on CT scan	Class 2	19	38.8
	Class 3	30	61.2
Patient position	Lateral	26	53.1
	Prone	23	46.9
Infection	Yes	1	2.0
	No	48	98.0
Associated injuries	Yes	18	36.7
	No	31	63.3
Reoperation	Yes	2	4.9
	No	39	95.1
Wound complications	Yes	4	8.3
	No	44	91.7

Variable	Mean	SD	Range
Age	31.12	7.50	30
Anesthesia time (minutes)	164.18	43.87	210
Operative time	128.47	38.42	200
Preoperative Bohler angle	9.33	13.07	86
Postoperative Bohler angle	22.69	9.15	45
Preoperative angle of Gissane	130.45	26.98	105
Postoperative angle of Gissane	124.76	17.20	67

### Differences between Lateral and Prone Position in the Characteristics and the Outcomes

The patient’s mean age was significantly higher in the lateral position group (33.31 ± 7.85) than the prone group (28.65 ± 6.37). Moreover, there was significant difference in the sanders classification between the two groups. The lateral position group had higher number of type III fracture (76.9%) when compared to the prone position (43.5%). Additionally, the frequency of associated injuries, as well as the mean preoperative Bohler’s and Gissane’s angles, were significantly higher in the lateral group. Also, the mean postoperative Bohler’s angle and angle of Gissane were significantly higher among the lateral group (25.88 ± 6.62, 137.15 ± 11.17) when compared to the prone one (19.09 ± 10.35, 110.74 ± 10.81), respectively (Table 2).

**Table 2:** Differences between lateral and prone positions

Variable		Lateral (*n* = 29)	Prone (*n* = 23)	*P* value
Sex	Females	2 (7.7)	4 (17.4)	0.239
	Males	24 (92.3)	19 (82.6)	
Mechanism of injury	FFH	25 (96.2)	20 (87.0)	0.455
	Others	1 (3.8)	3 (13.0)	
Laterality	Left	14 (53.8)	9 (39.1)	0.293
	Right	11 (42.3)	14 (60.9)	
	Bilateral	1 (3.8)	0 (0.0)	
Sanders classification	Class 2	6 (23.1)	13 (56.5)	0.035
	Class 3	20 (76.9)	10 (43.5)	
Infection	Yes	0 (0.0)	1 (4.3)	0.659
	No	26 (100.0)	22 (95.7)	
Associated injuries	Yes	15 (57.7)	3 (13.0)	0.004
	No	11 (42.3)	20 (87.0)	
Reoperation rate	Yes	1 (5.6)	1 (4.3)	0.947
	No	17 (94.4)	22 (95.7)	
Wound complications	Yes	2 (7.7)	2 (9.1)	0.773
	No	27 (92.3)	20 (90.9)	

Variable	Lateral (*n* = 29)	Prone (*n* = 23)	*P* value
Age	33.31 ± 7.85	28.65 ± 6.37	0.028
Anesthesia time	167.88 ± 54.11	160.00 ± 28.92	0.522
Operative time	130.58 ± 46.38	126.09 ± 27.67	0.688
Preoperative Bohler angle	13.81 ± 4.71	4.26 ± 17.23	0.016
Preoperative angle of Gissane	152.54 ± 7.79	105.48 ± 17.06	0.000
Postoperative Bohler angle	25.88 ± 6.62	19.09 ± 10.35	0.010
Postoperative angle of Gissane	137.15 ± 11.17	110.74 ± 10.81	0.000

There was no significant difference between the two groups regarding the reoperation rate (*p* 0.947), infection (*p* 0.659, operative time (*p* 0.688), anesthesia time (*p* 0.522) and wound complications (*p* 0.773).

### Factors Associated with Postoperative Bohler’s Angle and Angle of Gissane

The multivariable logistic regression analysis for the factors associated with postoperative Bohler Angle showed that only preoperative Bohler angle was significantly associated with postoperative Bohler Angle (Table 3; Adjusted B = 0.35; 95%CI: 0.16–0.55). Additionally, the multivariable regression analysis for the factors associated with postoperative angle of Gissane revealed that only patients’ position during the operation was significantly associated with postoperative angle of Gissane as prone position was significantly associated with reduction in postoperative angle of Gissane (Table 4; Adjusted B =  − 21.80; 95%CI: − 37.41– − 6.18).

**Table 3:** Factors associated with postoperative Bohler Angle

Variable	Adjusted B	(95%CI)
Sanders classification	2.31	(− 2.87–7.48)
Associated injuries	− 0.42	(− 4.98–5.82)
Age	− 0.12	(− 0.450–0.21)
Preoperative Bohler angle	0.35	(0.16–0.55)
Preoperative angle of Gissane	0.02	(− 0.16–0.20)
Patients position	− 1.96	(− 12.79–8.88)

**Table 4:** Factors associated with postoperative angle of Gissane

Variable	Adjusted B	(95%CI)
Sanders Classification	1.87	(− 5.59–9.32)
Associated Injuries	− 1.55	(− 9.33–6.23)
Age	− 0.05	(− 0.52–0.43)
Preoperative Bohler Angle	0.21	(− 0.07–0.49)
Preoperative Angle of Gissane	0.06	(− 0.20–0.32)
Patients Position	− 21.80	(− 37.41– − 6.18)

## Discussion

The most important finding of the present study is that there was a significant difference between the two groups with better restoration of the Gissane’s and Bohler’s angles in patients undergoing open reduction and internal fixation in the lateral decubitus position compared to prone position. However, there was no significant difference between the two groups regarding reoperation rate, infection, operative time, anesthesia time and wound complications.

Treatment of calcaneus fracture remains controversial, because of the suboptimal results and the high incidence of complications associated with both non-operative as well as operative treatments. On one hand, non-operative treatment is associated with higher percentage of joint arthritis, chronic pain and stiffness; on the other hand, surgical treatment is associated with higher incidence of wound complications and neurovascular injuries [4, 11, 14, 18–25].

The surgical aim of calcaneus fracture fixation is restoration of the articular facets, calcaneal height as well as the heel width. This can be achieved by restoration of the Bohler’s angle which asses the degree of joint depression and loss of height, and the Gissane’s angle which assess the relation between the calcaneal facets [26].

Loucks and Buckley evaluated the correlation of Bohler’s angle with surgical reduction of calcaneal fractures and concluded that surgical reduction improves the angles values and the patients function [27].

There have been no previous reports of comparing these two angles in patient undergoing calcaneal fixation in prone position versus lateral position.

Hasan et al. has reported significant restoration of the Gissane’s and Bohler’s angles with prone position [17].

Another finding of this study was a low incidence of infection (2%) and wound dehiscence (8%) compared to what has been reported in literature. The incidence of superficial infection has been reported to be as high as 27%, whereas deep infection was reported between 1.3 and 2.5% [4, 16].

This study had several limitations such as the retrospective design of the study and its associated biases. Additionally, multiple surgeons were involved in the operations. Moreover, the small number of cases, failure to report functional outcomes and lack of long term postoperative complications add to the limitations of this study. Therefore, a larger prospective stud can be undertaken in the future to avoid these limitations.

## Conclusion

Surgical restoration of the Bohler’s and Gissane’s angles with the patient placed in the lateral decubitus position remains superior to the prone position and is associated with better restorations of the angles. However, there was no difference in reoperation rate, infection, operative and anesthesia time and wound complications between the two groups.
